# New Resveratrol Oligomer Derivatives from the Roots of *Rheum lhasaense*

**DOI:** 10.3390/molecules18067093

**Published:** 2013-06-18

**Authors:** Wen-Bo Liu, Lin Hu, Qun Hu, Na-Na Chen, Qing-Song Yang, Fang-Fang Wang

**Affiliations:** 1 Key Laboratory of Chemistry in Ethnic Medicinal Resources, State Ethnic Affairs Commission & Ministry of Education, Yunnan University of Nationalities, Kunming 650031, Yunnan, China; E-Mails: liuwenbokm@126.com (W.-B.L.); chennana1111@126.com (N.-N.C.); smkms@126.com (Q.-S.Y.); foreverfang98@126.com (F.-F.W.); 2 Kunming Xianghao Technology Co., Ltd., Kunming 650204, Yunnan, China; E-Mail: huqun871@163.com

**Keywords:** *Rheum lhasaense* A*.* J. Li et P. K. Hsiao, resveratrol oligomers, rheumlhasol A, rheumlhasol B, DPPH radical

## Abstract

Two new resveratrol trimer derivatives, named rheumlhasol A (**1**) and rheumlhasol B (**2**) were isolated from the methanolic extract of roots of *Rheum lhasaense* A. J. Li et P. K. Hsiao together with four known resveratrol dimer derivatives, including maximol A (**3**), gnetin C (**4**), ε-viniferin (**5**), and pallidol (**6**). The structures were determined by combined spectroscopic methods and by comparison of their spectral data with those reported in the literature. All the compounds isolated from *R. lhasaense* were tested for their ability to scavenge1,1-diphenyl-2-picrylhydrazyl (DPPH) radical.

## 1. Introduction

Natural resveratrol oligomers, commonly consisting of two to eight resveratrol units, have drawn increasing attention across the world due to their intriguing structures and pharmacological potential [1,2,3]. Resveratrol oligomers provide dazzling chemical diversities with regard to the degree and pattern of polymerization, as well as their stereochemistry [4]. Most of them possess antioxidant activities because they have polyphenol functions in the molecules and are considered to be promising new sources of natural antioxidants [5,6]. However, resveratrol oligomers have been isolated from a relatively small assemblage of plant families. Vitaceae, Diterocarpaceae, Gnetaceae and Fabaceae provide a significant number of oligostilbenes [7,8,9].

The genus *Rheum* *Linn* consists of approximate 60 species and is mainly distributed in sub-alpine and alpine zones of Asia [10]. The underground part of *Rheum* spp. is commonly known as Da-Huang (rhubarb), and is used in traditional medicine for the treatment of constipation, inflammation, cancer, renal failure, and infectious diseases [11,12].

*Rheum lhasaense* A. J. Li et P. K. Hsiao is a stout herb primarily confined to the mountainous areas of eastern Tibet and adjacent regions [13]. The rhizomes and roots of this plant are locally known as “Qu Zha” and are traditionally used to help soothe the stomach (stomachic). 

Previous phytochemical investigation on *R. lhasaense* mainly focused on the analysis of anthraquinones, one of the most common and abundant substances in the roots of *Rheum* plants [14]. Surprisingly, *R. lhasaense* is very different from other species because of the absence of anthraquinones. No biological study on this special rhubarb has been conducted. A preliminary 1,1-diphenyl-2-picrylhydrazyl (DPPH) radical scavenging assay conducted by us demonstrated strong antioxidant activities in the methanolic extract of *R. lhasaense*. Therefore, the present study was carried out to investigate the bioactive constituents present in the medicinally important part of *R. lhasaense* plant.

Herein, we report the isolation and identification of two new resveratrol trimer derivatives named as rheumlhasol A (**1**) and rheumlhasol B (**2**) from the roots of *R. lhasaense*, together with four known resveratrol dimer derivatives, including maximol A (**3**), gnetin C (**4**), *ε*-viniferin (**5**), and pallidol (**6**). The antioxidant activities of all the isolated compounds were evaluated by the DPPH free radical-scavenging assay.

## 2. Results and Discussion

### 2.1. Structural Elucidation of the New Compounds

**Figure 1:**
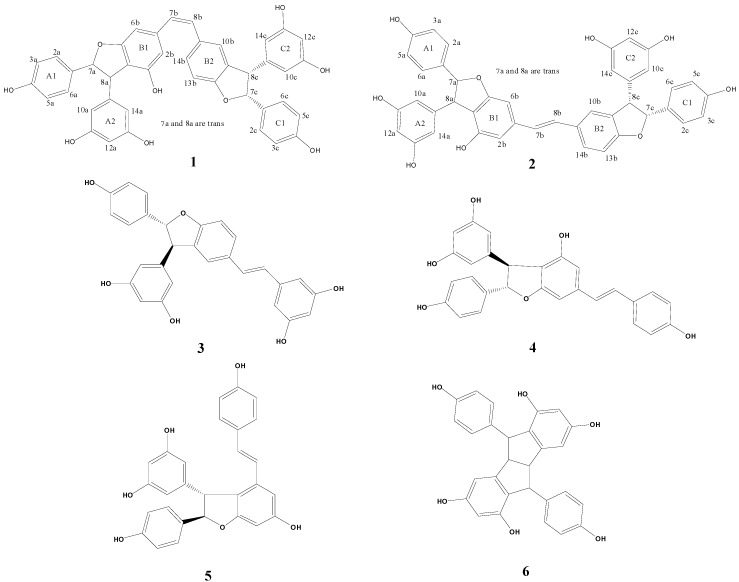
Chemical structures of compounds **1**–**6**.

The isolated compounds were identified by different spectroscopic analyses, including the extensive use of 1D (^1^H and ^13^C) and 2D NMR techniques (H−H COSY, HMBC, HMQC, and NOESY), and by comparing the experimental NMR data to the values reported in literature. The structures of isolated compounds are shown in Figure 1.

Rheumlhasol A (**1**) was isolated as a yellow amorphous powder. The molecular formula, deduced to be C_42_H_32_O_9_ by negative HR-ESI-MS ([M−H]^−^ at *m/z* 679.1964, calcd for C_42_H_31_O_9_), fitted well for a resveratrol trimer. The ^1^H-NMR spectra of **1** (Table 1) showed two sets of A_2_B_2_-type signals [δ_H_ ppm 7.12 (d, *J* = 8.5 Hz, 2H)_,_ and 6.77 (d, *J* = 8.5 Hz, 2H); 7.16 (d, *J* = 8.5 Hz, 2H), and 6.75 (d, *J* = 8.5 Hz, 2H)] and two sets of AX_2_-type signals [δ_H_ ppm 6.10 (d, *J* = 2.0 Hz, 2H), and 6.14 (t, *J* = 2.0 Hz, 1H); 6.10 (d, *J* = 2.0 Hz, 2H), and 6.17 (t, *J* = 2.0 Hz, 1H)] that were assigned to two *p-*substituted phenyl moieties (A1 and C1 rings) and two 1,3,5-trisubstituted aromatic rings (A2 and C2 rings), respectively, characteristic of the two resveratrol structural units. The presence of two sets of mutually coupled methine hydrogen signals [each set containing two deshielded oxymethine signals: δ_H_ ppm 5.28 (d, *J* = 5.4 Hz, 1H), and 4.31 (d, *J* = 5.4 Hz, 1H); 5.36 (d, *J* = 8.3 Hz, 1H), and 4.36 (d, *J* = 8.3 Hz, 1H)] instead of the olefinic proton signals suggesting that the olefinic bond got reduced thereby resulting in trimerisation of these carbons in the two resveratrol structural units. Furthermore, the ^1^H-NMR spectra of **1** displayed two signals [δ_H_ ppm 6.40 (d, *J* = 12.2 Hz, 1H), and 6.46 (d, *J* = 12.2 Hz, 1H)] that were assigned to the *cis*-coupled olefinic protons in the third resveratrol structural unit. Two aromatic rings (e.g., B1 and B2) of resveratrol structural units took part in the trimerisation process, as was evident from the ^1^H-NMR signals as follows: two singlets for *m*-hydrogens resonating at δ_H_ ppm 6.34 (s, 1H) and 6.32 (s, 1H) were assigned to a 1, 3, 4, 5-tetrasubstituted ring (B1), and two doublets for protons resonating at δ_H_ ppm 6.74 (d, *J* = 8.0 Hz, 1H), and 7.21 (d, *J* = 8.0 Hz, 1H) together with a singlet at δ_H_ ppm 6.94 (br s, 1H) were assigned to a 1, 3, 4-trisubstituted ring (B2).

**Figure 2:**
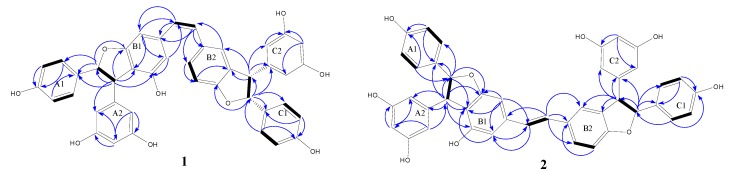
Main HMBC (indicated by blue arrows from ^1^H to ^13^C) and H-H COSY correlations (indicated by bold lines) of compounds **1** and **2**.

Further, with the aid of H−H COSY, HMBC, and HMQC NMR techniques, the chemical shift values were assigned and the structural connections between the three resveratrol units were established. In the HMBC spectrum of **1** (Figure 2): the correlations of H-8a with C-3b, C-4b, and C-5b; H-8c with C-10b, C-11b and C-12b; and H-10b with C-8c clearly revealed that unit A is connected to B1 ring through C-8a/C-4b, and unit C is connected to B2 ring through C-8c/C-11b. Furthermore, the presence of two dihydrofuran rings (e.g., 7a-8a-4b-5b-O and 7c-8c-11b-12b-O) was deduced by calculating the degrees of unsaturation and confirmed by the correlation of the cross-peaks: H-7a/C-5b and H-7c/C-12b in the HMBC spectrum.

**Table 1:** ^1^H, ^13^C-NMR and ROSEY (500M Hz) data of **1** and **2** (CD_3_OD, *δ* in ppm).

Positions	1			2		
	*δ*_H_ (mult, *J* in Hz, I)	*δ*_C_	ROSEY	*δ*_H_ (mult, *J* in Hz, I)	*δ*_C_	ROSEY
1a		134.2			134.3	
2a(6a)	7.12 (d, 8.5, 2H)	128.1	7a, 8a	7.13 (d, 8.5, 2H)	128.1	7a, 8a
3a(5a)	6.77 (d, 8.5, 2H)	116.3		6.76 (d, 8.5, 2H)	116.3	
4a		158.5			158.4	
7a	5.28 (d, 5.4, 1H)	94.5		5.30 (d, 5.4, 1H)	94.5	
8a	4.31 (d, 5.4, 1H)	56.7		4.33 (d, 5.4, 1H)	56.6	
9a		146.5			146.6	
10a(14a)	6.10 (d, 2.0 Hz, 2H)	107.1	7a, 8a	6.13 (d, 1.7 ,2H)	107.0	7a, 8a
11a(13a)		159.6			159.6	
12a	6.14 (t, 2.0 Hz, 1H)	102.0		6.16 (t, 1.7, 1H)	102.0	
1b		141.2			141.5	
2b	6.34 (s, 1H)	110.1		6.50 (s, 1H)	108.0	
3b		155.5			155.6	
4b		115.1			115.4	
5b		163.0			163.3	
6b	6.32 (s, 1H)	102.5		6.60 (s, 1H)	99.6	
7b	6.40 (d, 12.2, 1H)	129.8	8b	6.85 (d, 16.2, 1H)	127.4	
8b	6.46 (d, 12.2, 1H)	131.0	7b	7.02 (d, 16.2, 1H)	129.3	
9b		131.7			132.3	
10b	6.94 (br s, 1H)	127.4		7.20 (br s, 1H)	124.1	
11b		131.7			132.4	
12b		160.4			160.9	
13b	6.74 (d, 8.0, 1H)	109.8		6.84 (d, 8.3, 1H)	110.3	
14b	7.21 (d, 8.0, 1H)	130.6		7.37 (d, 8.3, 1H)	128.7	
1c		132.9			132.8	
2c(6c)	7.16 (d, 8.5, 2H)	128.7	7c, 8c	7.17 (d, 8.5, 2H)	128.7	7c, 8c
3c(5c)	6.75 (d, 8.5, 2H)	116.3		6.79 (d, 8.5, 2H)	116.3	
4c		158.7			158.6	
7c	5.36 (d, 8.3, 1H)	94.7	8c	5.39 (d, 8.4, 1H)	94.9	8c
8c	4.36 (d, 8.3, 1H)	58.7	7c, 10b	4.40 (d, 8.4, 1H)	58.7	7c, 10b
9c		145.4			145.3	
10c(14c)	6.10 (d, 2.0, 2H)	107.7	7c, 8c	6.15 (d, 1.7, 2H)	107.9	7c, 8c
11c(13c)		159.8			159.8	
12c	6.17 (t, 2.0, 1H)	102.5		6.22 (t, 1.7, 1H)	102.5	

**Figure 3:**
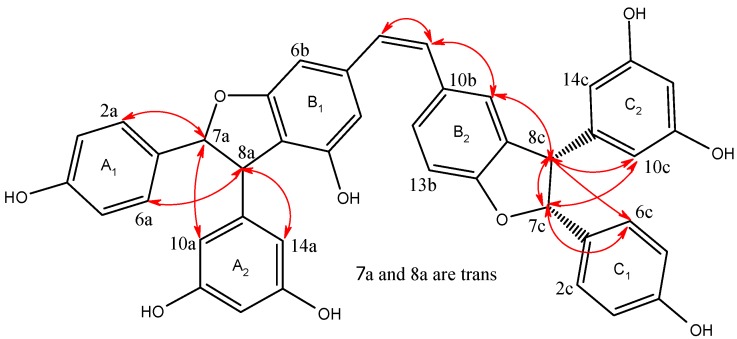
Main ROSEY (indicated by red arrows) of compound **1**.

The relative stereochemistry of the two dihydrofuran rings was assigned by the ROESY correlations (Table 1, Figure 3). Significant NOE interactions between H-7a/H-10a(14a) protons on A2 benzene ring and H-8a/H-2a(6a) protons on A1 benzene ring suggested that H-7a/H-8a protons are situated in a *trans*-orientation, which was confirmed by comparing the value of coupling constant (e.g., *J* = 5.4 Hz) to that of related resveratrol oligomers reported in literature [15,16,17]. Significant NOE interactions between H-7c/H-8c protons suggested that H-7c/H-8c protons are situated in a *cis*-orientation. However, no NOE interactions between either H-7c/H-7a(8a) or H-8c/H-7a(8a) protons were observed in the ROESY experiment due to their remote distance, and therefore the complete relative stereochemistry of **1** could not be assigned.

The molecular formula C_42_H_32_O_9_ for rheumlhasol B (**2**) was deduced from negative HR-ESI-MS ([M−H]^−^ at *m/z* 679.1943, calcd for C_42_H_31_O_9_). The ^1^H-NMR and ^13^C-NMR data (Table 1) of **2** are very similar to those of **1**, except for the appearance of two new signals [δ_H_ ppm 6.85 (d, *J* = 16.2 Hz, 1H), and 7.02 (d, *J* = 16.2 Hz, 1H)] with relatively low-field chemical shifts and large coupling constants owing to *trans*-olefinic coupling (instead of *cis*-olefinic coupling). Thus, rheumlhasol B (**2**) was characterized as the (*E*)-geometrical isomer of rheumlhasol A (**1**) and from these results, the structure of **2** was determined as shown in Figure 1. The remaining known compounds **3**–**6** were identified by comparison of their spectroscopic data with literature data.

### 2.2. Antioxidant Activities by DPPH Scavenging Capacities

The resveratrol oligomers **1**–**6** isolated from *R. lhasaense* were screened for their antioxidant activities by DPPH free radical-scavenging assay that has been widely used for the evaluation of antioxidant activities of natural products. The results obtained in this study are summarized in Table 2. Among these compounds, **2** and **3** exhibited relatively high antioxidant activities with IC_50_ values of 28.7 and 31.3 µM, respectively, which was comparable to that of *α*-tocopherol; while **1**, **4**, and **5** showed moderate activities with IC_50_ values in the range of 49.7 to 69.8 µM. Compound **6** showed lowest antioxidant activity with IC_50_ values of 190.2 µM.

**Table 2:** Antioxidant Activities of the Compounds **1**–**6**.

Compds.	DPPH radical IC_50_ (μM) ^a^
**1**	49.7 ± 2.3
**2**	31.3 ± 1.5
**3**	28.7 ± 1.0
**4**	69.8 ± 2.3
**5**	52.6 ± 1.1
**6**	190.2 ± 3.8
Vitamin E	27.9 ± 0.9

^a^ IC_50_ values were expressed as means ±standard deviation.

## 3. Experimental

### 3.1. General

The ^1^H-, ^13^C-, and 2D NMR spectra were recorded on Bruker DRX-500 (500 MHz) spectrometer with TMS as internal standard. The ESI-MS (negative ion mode) and HR-ESI-MS (negative ion mode) spectra were recorded on VG AutoSpe 3000 and API Qstar P ulsar LC/TOF spectrometers, respectively. The UV spectra were measured by using a Shimadzu double-beam 210A spectrophotometer. The IR spectra were recorded on a Bio-Rad FTS-135 spectrometer, in KBr pellets. The optical rotations were measured by using a SEPA-3000 automatic digital polarimeter. The column chromatographic separations were performed on silica gel (200–300 mesh size; Qingdao Marine Chemical Inc., Qingdao, China), or Lichroprep RP-18 gel (40–63 µm mesh size; Merck, Darmstadt, Germany). The column fractions obtained were monitored by TLC, and spots were visualized by heating the silica gel plates after spraying with 15% H_2_SO_4_ in water. The TLC and PTLC separations were performed on silica gel Gf 254 pre-coated plates (Qingdao Marine Chemical Inc.). 

### 3.2. Plant Materials

*R. lhasaense* A. J. Li et P. K. Hsiao plant materials were collected in August 2010 from LhaSa, Tibet Autonomous Region, China, and authenticated by Professor Anjen Li of Institute of Botany, Chinese Academy of Sciences. A voucher of the specimen (No. 2004080203) collected was deposited at School of Chemistry & Biotechnology, Yunnan University of Nationalities.

### 3.3. Extraction and Isolation of the Compounds

The air-dried powder roots (1 kg) of *R. lhasaense* A. J. Li et P. K. Hsiao were extracted exhaustively with 95% aqueous EtOH (5 × 10 L) at room temperature . The EtOH extract was concentrated *in vacuo* to yield a brown residue (200 g), which was suspended in water (200 mL), and extracted with EtOAc (3 × 200 mL). The combined organic phase was concentrated to yield a residue (89 g), which was loaded on a silica gel (SiO_2_) column (2 kg) and eluted with petroleum ether (PE)/acetone gradient to give five fractions (1–5). Fraction 3 eluted with PE/acetone (2:1) was subjected to repeated column chromatography (CC) (SiO_2_; CHCl_3_/MeOH, 10:1) to afford **3** (25 mg). Fraction 4 eluted with PE/acetone (1:2) was subjected to repeated CC (SiO_2_; CHCl_3_/MeOH, 10:1–8:2), followed by PTLC (CHCl_3_/MeOH, 9:1) to afford **4** (27 mg) and **5** (15 mg). Fraction 5 eluted with acetone was subjected to repeated CC (SiO_2_; CHCl_3_/MeOH, 10:1–5:1) to afford **6** (30 mg) and a sub-fraction containing **1** and **2**. This sub-fraction was subjected to repeated CC on RP_18_ gel eluted by MeOH/water (58:42) to afford **1** (10 mg) and **2** (12 mg). 

### 3.4. Spectroscopic Data

*Rheumlhasol A* (**1**): white powder; [α]_D_ = +10.2262^ο^ (*c* = 0.0056, MeOH); IR (KBr) *ν*_max_ 3419, 1603, 1515, 1486, 1449, 1339, 1301, 1233, 1155, 998, and 831 cm^−1^; UV (MeOH) *λ*_max_ (log *ε*) 202 (4.9), 227 (3.3), 285 (2.4), and 300 (2.2) nm; negative ESI-MS [M−H]^−^ at *m/z* 679; negative HR-ESI-MS [M−H]^−^ at *m/z* 679.1964 (calcd for C_42_H_31_O_9_ 679.1968); ^1^H and ^13^C-NMR data (Table 1).

*RheumlhasolB* (**2**): white powder; [α]_D_ = +5.4321^ο^ (*c* = 0.0054, MeOH); IR (KBr) *ν*_max_ 3396, 1600, 1516, 1486, 1450, 1341, 1303, 1235, 1155, 999, 960, and 832; UV (MeOH) *λ*_max_ (log *ε*) 202 (4.9), 225 (3.3), 310 (2.3), and 335 (2.4) nm; negative ion ESI-MS [M−H]^−^ at *m/z* 679; negative ion HR-ESI-MS *m/z* 679.1943 (calcd for C_42_H_31_O_9_ 679.1968); ^1^H and ^13^C-NMR spectra (Table 1).

*Maximol A* (**3**): brown amorphous powder, positive ESI-MS [M]^+^ at *m/z* 454; ^1^H-NMR (500 MHz, acetone-*d_6_*) δ ppm 7.43 (d, *J* = 8.3 Hz, 1H, H-6′), 7.24 (overlapping signals, 3H, H-2, H-6, and H-2′), 7.06 (d, *J* = 16.3 Hz, 1H, H-7′), 6.90 (d, *J* = 16.3 Hz, 1H, H-8′), 6.85 (d, *J* = 8.3 Hz, 1H, H-5′), 6.78 (overlapping signals, 3H, H-3, H-5, and H-8′), 6.53 (d, *J* = 1.7 Hz, 2H, H-10′ and H-14′), 6.28 (t, *J* = 1.7 Hz, 1H, H-12), 6.25 (t, *J* = 1.7 Hz, 1H, H-12′), 6.19 (d, *J* = 1.7 Hz, 2H, H-10 and H-14), 5.45 (d, *J* = 8.0 Hz, 1H, H-7), and 4.46 (d, *J* = 8.0 Hz, 1H, H-8); ^13^C-NMR (125 MHz, acetone-*d_6_*) δ ppm 160.54 (C-4′), 159.72 (C-11 and C-13), 159.52 (C-11′ and C-13′), 158.40 (C-4), 145.15 (C-9), 140.70 (C-9′), 132.44 (C-1′), 132.14 (C-3′), 131.68 (C-1), 129.04 (C-7′), 128.56 (C-2 and C-6), 128.56 (C-5′), 127.15 (C-8′), 123.86 (C-2′), 116.12 (C-3 and C-5), 110.11 (C-5′), 107.37 (C-10 and C-14), 105.64 (C-10′ and C-14′), 102.68 (C-12′), 102.34 (C-12), 94.00 (C-7), and 57.77 (C-8). These data are consistent with those reported in literature [18].

*Gnetin C* (**4**): yellow powder; negative ESI [M−H]^−^ at *m/z* 453; ^1^H-NMR (500 MHz, CD_3_OD) δ ppm 7.35 (d, *J* = 8.4 Hz, 2H, H-2 and H-6), 7.16 (d, *J* = 8.4 Hz, 2H, H-2′ and H-6′), 7.01 (d, *J* = 16.2 Hz, 1H, H-7), 6.89 (d, *J* = 16.2 Hz, 1H, H-8), 6.80 (overlapped signals, 4H, H-3, H-5, H-3′, and H-5′), 6.67 (br s, 1H, H-14), 6.58 (br s, 1H, H-10), 6.26 (t, *J* = 1.7 Hz, 1H, H-12′), 6.21 (d, *J* = 1.7 Hz, 2H, H-10′ and H-14′), 5.35 (d, *J* = 5.4 Hz, 1H, H-7′), 4.41 (d, *J* = 5.4 Hz, 1H, H-8′); ^13^C-NMR (125 MHz, CD_3_OD) δ ppm 163.36 (C-5′), 159.54 (C-11 and C-13), 158.27 (C-4), 158.09 (C-14), 155.57 (C-3′), 146.71 (C-9), 141.85 (C-1′), 134.31 (C-9′), 130.63 (C-1), 129.56 (C-7′), 129.11 (C-10′ and C-14′), 128.4 (C-2 and C-6), 127.15 (C-8′), 116.6 (C-3 and C-5), 116.51 (C-11′ and C-13′), 115.42 (C-4′), 108.15 (C-2′), 107.40 (C-10 and C-14), 102.26 (C-12), 99.94 (C-6), 94.64 (C-7), and 56.67 (C-8). These data are in accordance with those reported in literature [19].

*ε-Viniferin* (**5**): pale white powder; positive ESI [M+H]^+^ at *m/z* 455; ^1^H-NMR (400 MHz, CD_3_OD) δ ppm 7.14 (d, *J* = 8.4 Hz, 2H, H-2′ and H-6′), 7.04 (d, *J* = 8.4 Hz, 2H, H-2 and H-6), 6.82 (d, *J* = 16.3 Hz, 1H, H-7′), 6.77 (d, *J* = 8.4 Hz, 2H, H-3′ and H-5′), 6.65 (d, *J* = 8.4 Hz, 2H, H-3 and H-5), 6.63 (d, *J* = 1.6 Hz, 1H, H-14′), 6.57 (d, *J* = 16.3 Hz, 1H, H-8′), 6.25 (d, *J* = 1.6 Hz, 1H, H-12′), 6.18 (t, *J* = 1.7 Hz, 1H, H-12), 6.16 (d, *J* = 1.7 Hz, 2H, H-10 and H-14), 5.36 (d, *J* = 6.6 Hz, 1H), and 4.34 (d, *J* = 6.7 Hz, 1H); ^13^C-NMR (125 MHz, CD_3_OD) δ ppm 162.73 (C-3′), 160.05 (C-11 and C-13), 159.75 (C-5′), 158.54 (C-12′), 158.40 (C-4), 147.35 (C-9), 136.90 (C-1′), 133.87 (C-1), 130.36 (C-9′), 130.31 (C-7′), 128.77 (C-2 and C-6), 128.21 (C-10′ and C-13′), 123.66 (C-8′), 120.04 (C-2′), 116.36 (C-3 and C-5), 116.29 (C-11′ and C-13′), 107.44 (C-10 and C-14), 104.30 (C-6′), 102.18 (C-12), 96.83 (C-4′), 94.83 (C-7), and 58.30 (C-8). These data are in accordance with those reported in literature [20,21].

*Pallidol* (**6**): white powder; positive ESI-MS [M+H]^+^ at *m/z* 455; ^1^H-NMR (500 MHz, CD_3_COCD_3_) δ ppm 6.97 (d, *J* = 8.5 Hz, 4H, H-2, H-6, H-2′, and H-6′), 6.69 (d, J = 8.5 Hz, 4H, H-3, H-5, H-3′ and H-5′), 6.61 (d, J = 2.0 Hz, 2H, H-10 and H-10′), 6.18 (d, J = 2.0 Hz, 2H, H-12 and H-12′), 4.55 (s, 2H, H-7 and H-7′), and 3.80 (s, 2H, H-8 and H-8′); ^13^C-NMR (125 MHz, CD_3_COCD_3_) δ ppm 159.30 (C-11 and C-11′), 156.30 (C-4 and C-4′), 155.28 (C-13 and C-13′), 150.24 (C-9 and C-9′), 137.69 (C-1 and C-1′), 128.98 (C-2, C-6, C-2′, and C-6′), 123.18 (C-14 and C-14′), 115.75 (C-3, C-5, C-3′, and C-5′), 103.26 (C-10 and C-10′), 102.43 (C-12 and C-12′), 60.45 (C-8 and C-8′), and 53.89 (C-7 and C-7′). These data are in accordance with those reported in literature [22].

### 3.5. DPPH Assays

Sample stock solutions (1 mM) were diluted to concentrations of 25, 50, 100, 150, 200, and 250 µM in methanol. One milliliter of DPPH methanol solution (500 µM, final concentration = 125 µM) was added to 3.0 mL of a MeOH solution of various sample concentrations. The mixtures were shaken vigorously and then kept in the dark at room temperature. After 30 min, the absorbance values were measured at 518 nm and converted into the percentage inhibition of DPPH (Ip) by using the following formula:

Ip = [ (Abs_sample_ − Abs_control_)/Abs_control_] × 100

A mixture of DPPH solution (1.0 mL, 160 µM) and methanol (3.0 mL) was used as negative control while *dl*-*α*-tocopherol solution was used as positive control. The IC_50_ values obtained represent the concentrations of the tested samples and standards that caused 50% inhibition of DPPH, and were calculated by linear regression of plots where the abscissa represent the concentration of tested compounds and the ordinate represent the average percentage of inhibition from three separate tests. The experiments were done in triplicate, and the results are given as mean ± standard deviation (SD).

## 4. Conclusions

Two new isomeric resveratrol trimers named rheumlhasol A (**1**) and rheumlhasol B (**2**) were isolated from the roots of *Rheum lhasaense* A. J. Li et P. K. Hsiao, together with four known dimers **3**−**6**. Apparently, compound **2** is derived from the coupling of gnetin C (**4**) with another resveratrol unit. The benzofuran ring connects B unit and C unit may formed from condensation of oxygen radical (C12-O^·^) and carbon C-11 of B2 ring with C-7c and carbon radical (C-8c) of C unit. This is the first time that *Rheum* plants have been reported to contain resveratrol trimers. In addition, the free radical scavenging activities of all the isolated compounds against DPPH radical have been evaluated in this study. Compounds **1**–**5** showed moderate antioxidant activities, with IC_50_ values in the range of 28.7 to 69.8 μM, while compound **6** showed low antioxidant activity with an IC_50_ value of 190.2 μM.
